# SeQuaiA: A Scalable Tool for Semi-Quantitative Analysis of Chemical Reaction Networks

**DOI:** 10.1007/978-3-030-53288-8_32

**Published:** 2020-06-13

**Authors:** Milan Češka, Calvin Chau, Jan Křetínský

**Affiliations:** 8 Microsoft Research Lab, Redmond, WA USA; 9 University of Southern California, Los Angeles, CA USA; 10 Brno University of Technology, Brno, Czech Republic; 11 Technical University of Munich, Munich, Germany

## Abstract

Chemical reaction networks (CRNs) play a fundamental role in analysis and design of biochemical systems. They induce continuous-time stochastic systems, whose analysis is a computationally intensive task. We present a tool that implements the recently proposed semi-quantitative analysis of CRN. Compared to the proposed theory, the tool implements the analysis so that it is more flexible and more precise. Further, its GUI offers a wide range of visualization procedures that facilitate the interpretation of the analysis results as well as guidance to refine the analysis. Finally, we define and implement a new notion of “mean” simulations, summarizing the typical behaviours of the system in a way directly comparable to standard simulations produced by other tools.

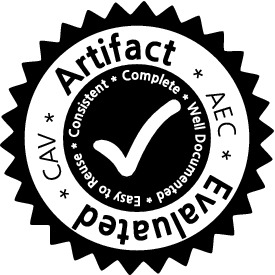



## Introduction

*Chemical Reaction Networks (CRNs)* are a language widely used for *modelling and analysis* of biochemical systems [10] as well as for high-level programming of molecular devices [6, 33]. They provide a compact formalism equivalent to Petri nets [30], vector addition systems [24] and distributed population protocols [3]. A CRN consists of a set of chemical reactions of given species, each running at a certain rate (intuitively, speed).

### Example 1

**Fig. 1.**
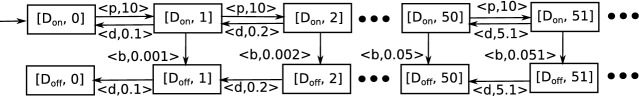
The Markov chain for Gene expression, displaying the population of P. To simplify the exposition, D$_\mathrm{on}$ and D$_\mathrm{off}$ are displayed as discrete “states” of the system, but in fact the two “states” are just shorthands for 1,0 and 0,1, respectively.

*(Gene expression).* Our running example is the classic simple expression of a protein given by the reactions of production (p) and degradation (d) of proteins and blocking (b) the DNA, over three species: protein (P), active DNA (DNA$_\mathrm{on}$), and blocked DNA (DNA$_\mathrm{off}$):
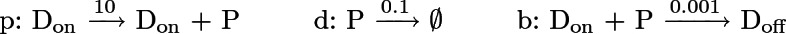


Using mass-action kinetics (the reaction rate is multiplied by the populations of the reactants), the CRN induces a infinite population Markov chain in Fig. 1.

In order to facilitate numerous applications in systems and synthetic biology, various techniques for simulation and formal analysis of CRNs have been proposed, e.g. [2, 7, 15, 18, 32]. We pinpoint several specifics of this setting, necessary to motivate and understand the features of the tool:

1. The analysis is notoriously difficult and **computationally expensive** due to several aspects: *state-space explosion* (exponential growth in the number of species, possibly infinite spaces due to unbounded populations as in Fig. 1, different rates for different populations, again as in Fig. 1), *stochasticity* (races between reactions), *stiffness* (rates of different magnitudes), *multimodality* (qualitatively different behaviours such as extinction of predators only, or also of preys in the predator-prey models) [17, 34]. Consequently, even for small CRNs, simulations may take minutes and analyses hours.
2. We have to face **imprecise inputs**. In particular, even if all relevant reactions are known, the rates are typically not. It is then not clear what behaviours can be induced by all possible values.
3. The analysis **output need not be precise** numerically, but only qualitatively. For instance, it is important to know that initial growth is followed by extinction and what the order of magnitude of the peak population is, but not necessarily what the exact distribution at an exact time is. Unfortunately, it is hard to compute the qualitative information without the quantitative one.
4. Biologists and engineers often seek for plausible **explanations** of why the system under study features or not the discussed behaviour. In many cases, a set of system simulations/trajectories or population distributions is not sufficient and the ability to provide an accurate explanation for the temporal or steady-state behaviour is another major challenge for the existing techniques.

*SeQuaiA*^1^ is a tool for analysis of CRN addressing these issues:

1. It features unprecedented **scalability**, analysing standard complex benchmarks within a fraction of a second.
2. It is **robust** w.r.t. concrete rates, not depending on the exact values but only on their orders of magnitude.
3. Its *semi-quantitative analysis* is **precise enough** to conclude on the qualitative behaviour of the system including rare behaviours and on rough estimates of the quantities (population sizes, times).
4. It produces small abstract models (Markov chains) that are explicit, yet **interpretable**, making the behaviour more **explainable**.

It is based on the technique presented in [9], relying on two cornerstones. Firstly, it computes a system abstraction with **acceleration**, abstracting not only states and single transitions, but taking into account *segments* of paths. The resulting models are small enough to allow for a synoptic observation of the model dynamics. Secondly, it performs **semi-quantitative analysis**, focusing on the most probable behaviours and more qualitative, global descriptions, such as oscillation, rather than fully quantitative sequences of exact transient distributions. This yields explainable models and is a sufficient and computationally cheaper technique. While the basic theory is derived from [9], there are a number of new features and differences in our tool, not just the implementation:

- **Method:** (i) The abstraction is *more precise* now that the tool can also compute numerical outputs, whereas [9] focuses on a manually feasible, and hence imprecise, abstraction. (ii) It suggests how to *refine the abstractions*, providing a knob for trading precision for computational resources.
- **Visualization:** The GUI provides a number of ways to display the results, facilitating understanding the models, including (i) identification of strongly connected parts of ‘iterations’, corresponding to ‘temporarily stable’ behaviours, (ii) quantitative information on transient times and steady-state distributions, or (iii) visual qualitative explanations, such as semantic grouping of states or tracking correlations between populations.
- **Additional analysis instruments:** (i) The new notion of *envelope* provides an explicit knob to consider not only the most probable, but also less probable behaviours. (ii) The novel concept of *mean simulation* yields summaries of most probable runs and an analysis output directly comparable to classic simulation-based tools.

*Related Work.* Since a direct analysis of the Markov chains induced by CRN does not scale well [19], deterministic approximations through fluid (mean-field) techniques can be applied [4, 8] to large populations, but cannot adequately capture the stochasticity of CRNs caused by low population species. To this end, both can be combined in hybrid approaches [7, 18, 21], typically involving a computationally demanding numerical analysis. Reduction techniques such as [1, 12] are based on approximate bisimulation [11], on aggregation according to the CRN-specific structure [13, 27, 35], or state truncation [20, 28, 29].

Despite the plethora of techniques, the practical analysis of CRNs often relies on the stochastic simulation [15] and its multi-scale improvements [5, 14, 17, 22, 31, 32]. The widely used tool include the platform-independent Copasi [23], DSD [25] with a convenient web-based graphical interface, or StochPy [26] easily extensible using Python scientific libraries. In contrast, our approach (i) provides a compact explanation of the system behaviour in the form of tiny models allowing for a synoptic observation (ii) can easily reveal less probable behaviours, and iii) as shown in [9], is able to analyse standard complex benchmarks in seconds and thus provides the unprecedented scalability compared to other numerical as well as simulation-based techniques.

## Workflow and Key Functionality

In this section, we guide the reader through the workflow, discuss the key features of the tool and demonstrate them on examples. The GUI is structured into several tabs and panels reflecting the workflow of the tool. First, a CRN is either retrieved from a file in the Open model tab or a new one is created. Either way, the model can be changed in the Editor panel together with the analysis parameters. The process continues in the Analysis tab. The analysis follows in two steps. First, the *semi-quantitative abstraction* of the Markov chain for the CRN is generated; second, the *semi-quantitative* analysis is performed on the abstraction. The tool offers an explicit option to display the abstraction as a .dot file or to directly run both steps. After the complete analysis is executed, the Visualization panel offers a range of options to *display* the results, including various *quantitative properties*. Finally, the analysed model can be used to generate concrete runs on the Simulation tab, which we call *mean simulations* since they display the “average-case” behaviour. In the following we detail on these key elements.

**Fig. 2.**
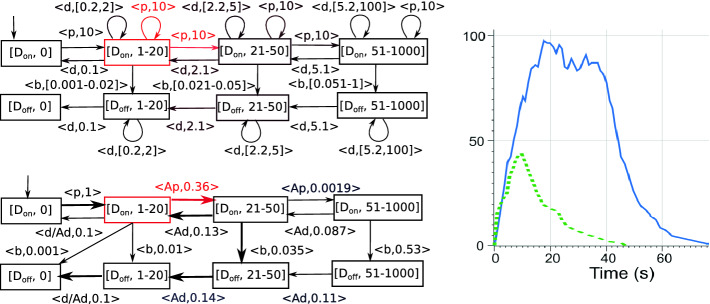
**Left:** The abstract Markov chain for Gene expression with population discretization thresholds 20, 50 and the population bound 1000. *Top:* The classic may transition function. *Bottom:* The semi-quantitative version with accelerated transitions (denoted by prefix “A”). **Right:** The full blue line shows a typical simulation of the model (population of P), obtained using DSD tool [25]. The dotted green line corresponds to the fast variant of the model with the rate of *b* being $10^{-2}$. (Color figure online)

### Semi-quantitative Abstraction

*Key Idea.* The abstraction of the state space is simply given by a discretization of the population for each species into finitely many intervals, see Fig. 2 (left). The classic may abstraction of the transition function results in non-deterministic self-loops as in Fig. 2 (left top) in red, which make impossible to conclude anything useful (except for some safety properties) on the behaviour once we reach such a state, even whether it is ever left at all. Instead, [9] considers sequences of transitions: in this case, sequences of prevalently growing transitions (those increasing the population) are significantly more probable than the prevalently decreasing ones. Consequently, the self-looping transitions are *accelerated* (taken multiple times) to get a “combined” transition that brings a typical representative of this population interval into a higher interval, see Fig. 2 (left bottom) also in red. Hence the new rate reflects (i) the mass-action kinetics with the typical population in the interval and (ii) the typical number of the transition repetitions before another interval is reached. These accelerated transitions are the key idea of the semi-quantitative abstraction and are denoted by a prefix *A*.

*Tool Inputs.* Technically, the tool requires, for each species, a (possible empty) list of increasing population thresholds $t_1,t_2, \ldots t_n$ and a population bound $t_b$. The thresholds split the concrete population to the intervals $[0,0],(0,t_1],(t_1,t_2],$ $\ldots (t_{n-1},t_n], (t_{n},\infty )$. Here 0 is taken separately to reflect enabledness of actions; the representatives, used for consequent computations, are chosen to be in the middle of the intervals and derived from $t_b$ for the last one. (For the empty list we have only one non-zero interval $(0,\infty )$). The input numbers are supposed to reflect the monitored property of interest and the required precision, the bound $t_b$ should give a probable upper bound on the maximal population. How to obtain and iteratively improve these is discussed in Sect. 2.5 on refinement.

#### Example 2

**Fig. 3.**
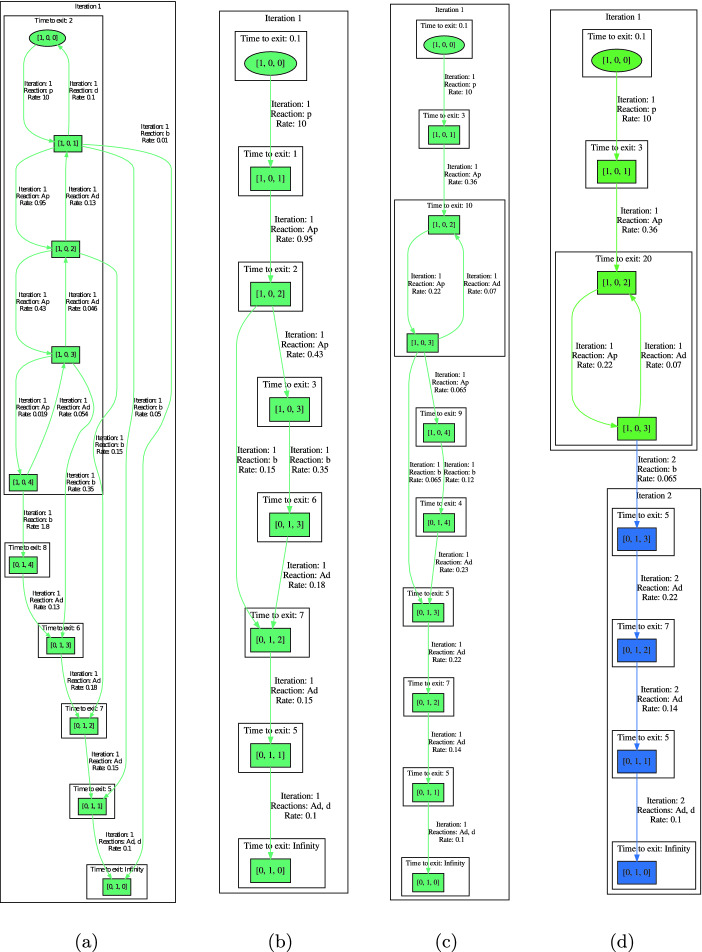
(a) and (b): ‘Fast’ Gene expression with thresholds 10, 20, 50. (a) depicts the full abstraction and (b) depicts $$ envelope =3$$. (c)–(e): ‘Slow’ Gene expression with thresholds 20, 50, 80, 150. (d) and (e) depicts the pruned abstraction with $$ envelope =3$$ and 1, respectively.

Consider Gene expression, now with a ‘fast’ blocking where the rate of *b* equals $10^{-2}$. A typical simulation can be seen in Fig. 2 (right, dotted green line): the number of proteins grows until several dozen, then blocking takes place until extinction. The semi-quantitative abstraction for thresholds 10, 20, 50 yields the model in Fig. 3(a). In contrast to classic abstractions, there are no self-loops and the abstract transitions are assigned concrete rates. One can see that the blocking can in principle take place at any population and that population can decrease also when DNA is on, i.e. in states $[1,0,\cdot ]$. However, all this happens with very low probabilities and the model captures this only indirectly through the numerical labelling. This is made explicit during the semi-quantitative analysis.

### Semi-quantitative Analysis

*Key Idea.* The aim is to prune the abstraction so that only reasonably probable behaviour is reflected, see the thick transitions in the abstraction in Fig. 2 (left bottom). To this end, we preserve in each state only the transitions with the highest rate *h* or almost highest rates, i.e. with $$h'>h / envelope $$ where $$ envelope >1$$ is a parameter. Parameter values in [1, 10] ensure we can only look at rates of the same order of magnitude, thus the most probable events and those with e.g. only 20% chance of happening. Higher values then allow for inspection of even less probable behaviours.

Consequently, the method can naturally handle uncertainty in the reaction rates since typically only the relative magnitudes of the rates are important, actually, only their orders of magnitude. This robustness w.r.t. the input is very beneficial for biologists as the precise rates are often not known.

#### Example 3

The analysis of the previous ‘fast’ Gene expression with $$ envelope =3$$ is depicted in Fig. 3(b). As such it shows the most probable behaviours: the fast growth until the intervals 2 and 3 (i.e. 10–20 and 20–50) and not beyond to 4 (over 50), followed by a slower decline. The computed rates induce expected times to pass through a state, matching closely those of the simulation Fig. 2 (right, dotted green line). Moreover, we see that the blocking transition from interval 2 has a lower probability than the production, is thus less probable. As such it would not even appear as a probable one, for a stricter $$ envelope =2$$.

#### Example 4

A more complicated behaviour arises when the blocking is slow, with rate $10^{-3}$ as in Sect. 1. A simulation run for this case is depicted in Fig. 2 (right, full blue line). One can observe a more balanced competition between blocking and oscillation around 70–100 proteins. Similarly, while the full abstraction (not shown here) features arbitrary oscillations (also back to no proteins at all), after analysis the pruned abstraction is faithfully modelling the initial growth, subsequent oscillation only in the range of higher populations, followed by blocking and gradual extinction of proteins, see Fig. 3(c).

Technically, the analysis relies on repeated alternation of transient and steady-state analysis. First, starting from the initial state, we follow in each state only the transitions with highest rates (most probable ones), until the set of explored state reaches a fixpoint. A part of the created graph is recurrent and forms a bottom strongly connected component (BSCC) or a collection thereof. The system temporarily settles in the steady state of this BSCC. After some time has passed, also a less probable transition happens almost surely and the “BSCC” is exited. These exit points are identified by a steady-state analysis of the BSCC, taking the magnitudes of exiting and non-exiting transition rates into account. The exit points trigger a new *iteration* of the transient and then the steady-state analysis.

#### Example 5

Figure 3(d) illustrates a situation with two iteration using the slow variant of the model. Decreasing $envelope$ to 1 caused that the blocking reaction is explored in the second iteration – as an exit of the BSCC found in the first iteration. Before that exit happens, the “BSCC” represents a “temporary” steady state of the system.

*Note on Correctness.* As discussed in [9], the semi-quantitative analysis provides guarantees in the form of limit behaviour and convergence: firstly, the precision grows with the differences in the orders of magnitudes of involved rates: as their ratios tend to infinity, the error tends to zero; secondly, as the population discretization gets finer, the error in the new “accelerated” transitions is reduced, trivially being zero for complete refinement into singletons.

### Visualization of Qualitative Information

A proper visualization is essential for clear presentation and easy interpretation of the results of our analysis. To this end, the tool and its GUI offer various options for visualizing the results. The basic ones, related to the graph structure, are the following. Further options, with more quantitative flavour, are discussed in the next section, followed by an example illustrating all of them.

*Iterations.* As the complete abstract model is typically very large and chaotic, further structuring is necessary. Therefore, the default view shows the states arranged and grouped into separate blocks, one for each iteration, additionally coloured distinctly for each iteration. Besides, we can restrict which iterations we show. This is useful to zoom in and investigate a particular part of the behaviour.

*Intra-iteration SCCs (IISCCs).* Additionally, the arrangement and colouring can be based on aggregating SCCs *within* each iteration (IISCCs). This helps to understand the emergence of repetitive behaviour patterns, such as oscillation or (temporary) steady state. It can be also combined with the iteration grouping.

*Collapsed Views.* In order to understand the system behaviour, one typically needs to have a synoptic overview of the system. For more complex systems, even the pruned abstraction could become too large and the view of the fully expanded system might not be sufficiently compact. In such cases, the aggregates discussed in the previous views, i.e., iterations and IISCCs, can be collapsed into a single nodes, hiding the complexity of the exact behaviour pattern within these areas. This allows us, for instance, to ignore the particular (temporary) oscillation or steady state in these states and to focus on more global behaviour, such as what happened before and after this behaviour and how often does it arise. In contrast to zooming in by restricting to certain iteration(s) only, the collapsed views provide a means to zoom out.

### Visualization of Quantitative Information

The produced graphs are also labelled by *numerical information*. While the quantities cannot be precise due to the simplifications of the extremely scalable analysis, they match the orders of magnitudes of the observed quantities, which is often precise enough for biological purposes; for instance, the peak of protein growth happens after units vs. dozens of seconds in the fast and slow variants of Gene expression, respectively.

*Transient Analysis.* Firstly, each abstract transition is labelled with a rate corresponding (in the order of magnitude) to the rate of the concrete transition (or accelerated transition, i.e. a “sequence” of transitions) of a “typical” representative of the abstract state. These rates induce the expected time spent in each transient state of each iteration. Indeed, the waiting time is simply the inverse of the sum of the outgoing rates. Further, each BSCC of each iteration is labelled by an estimate of time before it is left into the next iteration. This is a key notion, which allows us to easily provide transient timing information for very stiff systems (working at different time scales). Consider the simple gene model. From Fig. 3(b) and (d) we can easily compute the expected time to the extinction (as the sum of the exit time for all SCC on the inspected path). Our analysis correctly estimates that the expected extinction time is around 24 and for the fast variant and 40 for the slow variant.

*Steady State Analysis.* In many biological models, the natural steady state is either extinction or unbounded explosion. Hence it does not say much about the “seemingly steady” state (the temporary steady state), i.e., behaviour that is stable for a long but finite time. Therefore, the tool provides information not only on the steady state of the whole system, but also for each iteration separately since they represent the temporary steady states discussed above. Both can be visualized as colouring of states, with higher probabilities corresponding to darker colours, immediatelly giving a synoptic view on frequent behaviours.

*Correlations.* Finally, correlations between population sizes can be observed as follows. The GUI can be given a set of equivalences of the form $m\!\sim \! n$ for species *i*, *j*, meaning that if a state has (abstract) population *m* of species *i* and *n* of *j* then it is regarded as satisfying the correlation in question. It is coloured accordingly and the overall colouring of the system provides further indication under which behaviour or in which phases the correlation holds.

**Fig. 4.**
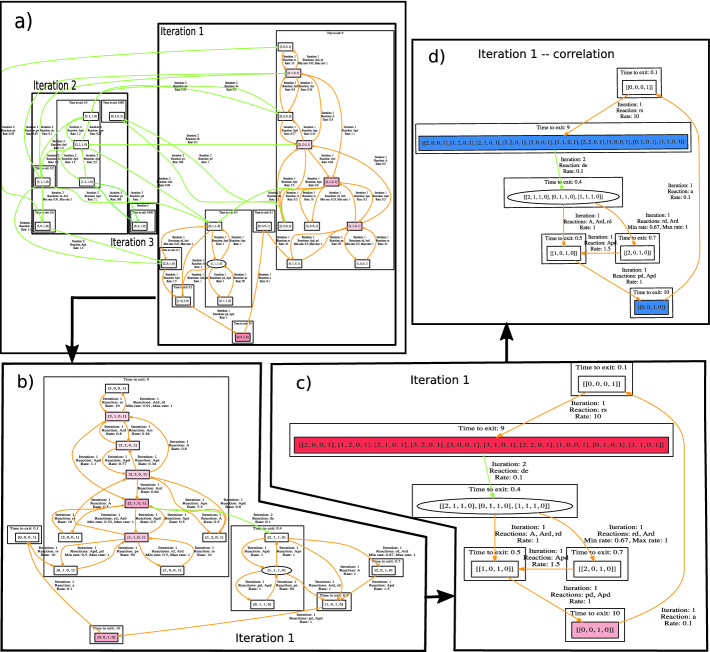
A visualisation of the workflow for the extended gene expression model. (Color figure online)

#### Example 6

We demonstrate these visualization options on a more complicated gene expression model [16], widely used model for benchmarking CRN analyzers, in Fig. 4. As reported in [16, 18], the behaviour oscillates between two steady states with DNA on and DNA off. Moreover, there is a correlation between high amounts of RNA present and DNA being on, and no RNA with DNA off.

The complete system and its steady state distribution is depicted in the part a) using the iteration and IISCC arrangement. This view shows immediately without seeing any details that the only interesting states are in iteration 1 including all states with a high steady-state probability (the red colouring). Therefore, in part b), we zoom in to iteration 1 and use the IISCC arrangement. In order to observe the interesting switches between the temporary steady states, we collapse the IISCCs, in the part c), and thus ignore the internal (non-interesting) behaviour of the big IISCC. Finally, in part d), we use the correlation colouring to identify states where the required correlation holds (i.e. the blue states). Comparing part c) and d) immediately reveals that the system spends the majority of the time in the states where the correlation holds.

### Precision and Refinement

So far, we have illustrated the concepts and the functionality on models with an appropriate level of abstraction. However, it often happens that we start the investigations with a too coarse abstraction. Whenever this happens, it is important to notice this and appropriately refine the abstraction. While [9] does not discuss this issue, the tool provides support also for that.

*Precision Parameters.* There are several knobs for trading the size and the precision of the abstraction. They all come as input in the lower half of the Editor tab: discretization, bound, and envelope.

#### Example 7

Recall the initial abstraction for the Gene expression of Fig. 2 (with rate $10^{-3}$). The abstraction, using thresholds 20, 50 predicts an oscillation including low populations of P (1–20) which is not correct (recall that the P oscillates on high populations before the blocking reaction occurs). Figure 3(c) and (d) show the abstraction and the consequent analysis and visualization for a refined model using thresholds 20, 50, 80, 150 (instead of just 20, 50). As already discussed, this abstraction already correctly predicts the system behaviour.

*Discretization.* The basic building block of each abstraction is the degree of details it preserves in the abstract states. Firstly, it determines how precisely we can observe the evolution of the population. For instance, whenever we want to detect whether a population typically grows beyond a bound or oscillates in a certain interval, such an interval should be present in the discretization. Secondly, the discretization should be fine enough so that in each state, the rates are reasonably (in orders of magnitude) precise. Fortunately, in our analysis their absolute precision is not vital. In contrast, we only need *relative* proportions of the rates to have the right *magnitude* to decide which behaviour is probable. Consequently, too rough abstraction is reflected in *“non-determinism”* when a state has two transitions under similar rate. In such a case, the probable behaviour cannot be determined. Therefore, the Visualization tab provides in the Colorization pane an option to provide suggestions for refinement, including highlighting non-deterministic states, pointing at the natural candidates for refinement. Note that we highlight only the states where the two transitions lead to mutually different SCCs so that a significant change in behaviour may occur.

*Bounds.* Similarly, for the single infinite interval $(t_n,\infty )$, the tool inputs a *bound* which is a believed safe upper bound on the population of the species. Of course, it may be wrong. This is irrelevant in case when the population explodes beyond all bounds. However, whenever there are transitions from the highest level back to a lower one, its feasibility and rate are in question. Optimally, such states do not even occur in the pruned abstraction. If they do, we also highlight them using the Colorization for Refinement suggestions (in another colour).

*Envelope.* As too rough abstractions introduce too much non-determinism, dually, the degree of the non-determinism is determined (even defined) by the *envelope*, the factor between rates so that even the less probable option is still taken into account (and thus introduces non-determinism). Consequently, high values of envelope introduce non-determinism, making the analysis take also less important behaviour into account; in contrast, low values make the analyzed system deterministic, showing only the most probable behaviour. The choice of the envelope thus depends on whether such behaviours should also be reported.

### Mean Simulations

Since our models, although abstract, have an operational semantics, we can even run simulations on them. Moreover, the accelerated transitions, as “sequences” of transitions, have a low variance in the expected time, by the law of large numbers. Hence their execution time can be chosen quite precisely in a deterministic way. Similarly, the time to leave an IIBSCC is quite deterministic. Thus we can generate simulation where the only random decisions are choices of transitions, but the timing follows the mean time of the respective events. Moreover, runs within the pruned abstraction reflect the most important behaviours only.

Such *mean simulations*^2^, which can thus be generated from our analysis, represent groups of typical runs (modulo small time shifts and order of transitions within an SCC, which are not very relevant). Therefore, a few such simulation reflect all the present behaviours (on a level of desired significant probability) and can serve to observe multi-modalities, bifurcations, rough transient timing as well as frequencies in the steady-state and temporary steady-state. To our best knowledge, such a concept has not yet been considered for simulation of stochastic systems.

**Fig. 5.**
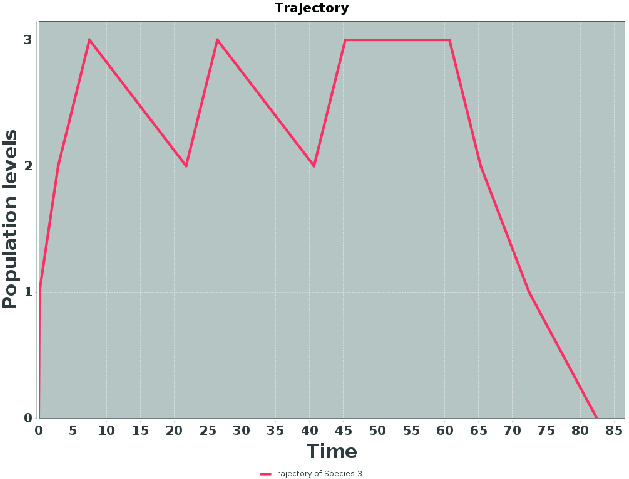
Mean simulation for the slow variant of Gene expression, directly comparable to Fig. 2 (right, full line).

#### Example 8

Figure 5 shows an abstract simulation for our running example with discretisation thresholds 20, 50, 80, 150. One can readily observe its validity with respect to the typical stochastic simulation in Fig. 2 (right, full blue line).

## Conclusion

We have presented SeQuaiA, a scalable tool for robust and explainable analysis of CRNs. The analysis is precise enough as cross-validated with simulation-based results on several models widely used in the literature. One of the key contributions of the tool is the visualization, which is essential for clear presentation and easy interpretation of the results of our analysis.

## Footnotes

1 Available at https://sequaia.model.in.tum.de.

2 They are not means of values from simulations since averaging oscillating values may result in no oscillation. Rather they reflect “mean patterns”.
